# Monitoring compliance with standards of care for chronic diseases using healthcare administrative databases in Italy: Strengths and limitations

**DOI:** 10.1371/journal.pone.0188377

**Published:** 2017-12-12

**Authors:** Rosa Gini, Martijn J. Schuemie, Alessandro Pasqua, Emanuele Carlini, Francesco Profili, Iacopo Cricelli, Patrizio Dazzi, Valentina Barletta, Paolo Francesconi, Francesco Lapi, Andrea Donatini, Giulia Dal Co, Modesta Visca, Mariadonata Bellentani, Miriam Sturkenboom, Niek Klazinga

**Affiliations:** 1 Agenzia regionale di sanità della Toscana, Osservatorio di epidemiologia, Florence, Italy; 2 Department of Medical Informatics, Erasmus Medical Center, Rotterdam, The Netherlands; 3 Janssen Research & Development, Epidemiology, Titusville, New Jersey, United States of America; 4 Observational Health Data Sciences and Informatics (OHDSI), New York, New York, United States of America; 5 Health Search, Italian College of General Practitioners and Primary Care, Florence, Italy; 6 Consiglio Nazionale delle Ricerche, Istituto di Scienza e Tecnologie dell'Informazione, Pisa, Italy; 7 Genomedics, Florence, Italy; 8 Assessorato Politiche per la Salute, Bologna, Italy; 9 Agenzia Nazionale per il Servizi Sanitari Regionali, Rome, Italy; 10 Academic Medical Center, University of Amsterdam, Amsterdam, The Netherlands; Universita degli Studi di Ferrara, ITALY

## Abstract

**Background:**

A recent comprehensive report on healthcare quality in Italy published by the Organization of Economic Co-operation and Development (OECD) recommended that regular monitoring of quality of primary care by means of compliance with standards of care for chronic diseases is performed. A previous ecological study demonstrated that compliance with standards of care could be reliably estimated on regional level using administrative databases. This study compares estimates based on administrative data with estimates based on GP records for the same persons, to understand whether ecological fallacy played a role in the results of the previous study.

**Methods:**

We compared estimates of compliance with diagnostic and therapeutic standards of care for type 2 diabetes (T2DM), hypertension and ischaemic heart disease (IHD) from administrative data (IAD) with estimates from medical records (MR) for the same persons registered with 24 GP’s in 2012. Data were linked at an individual level.

**Results:**

32,688 persons entered the study, 12,673 having at least one of the three diseases according to at least one data source. Patients not detected by IAD were many, for all three conditions: adding MR increased the number of cases of T2DM, hypertension, and IHD by +40%, +42%, and +104%, respectively. IAD had imperfect sensitivity in detecting population compliance with therapies (adding MR increased the estimate, from +11.5% for statins to +14.7% for antithrombotics), and, more substantially, with diagnostic recommendations (adding MR increased the estimate, from +23.7% in glycated hemoglobin tests, to +50.5% in electrocardiogram). Patients not detected by IAD were less compliant with respect to those that IAD correctly identified (from -4.8 percentage points in proportion of IHD patients compliant with a yearly glycated hemoglobin test, to -40.1 points in the proportion of T2DM patients compliant with the same recommendation). IAD overestimated indicators of compliance with therapeutic standards (significant differences ranged from 3.3. to 3.6 percentage points) and underestimated indicators of compliance with diagnostic standards (significant differences ranged from -2.3 to -14.1 percentage points).

**Conclusion:**

IAD overestimated the percentage of patients compliant with therapeutic standards by less than 6 percentage points, and underestimated the percentage of patients compliant with diagnostic standards by a maximum of 14 percentage points. Therefore, both discussions at local level between GP's and local health unit managers and discussions at central level between national and regional policy makers can be informed by indicators of compliance estimated by IAD, which, based on those results, have the ability of signalling critical or excellent clusters. However, this study found that estimates are partly flawed, because a high number of patients with chronic diseases are not detected by IAD, patients detected are not representative of the whole population of patients, and some categories of diagnostic tests are markedly underrecorded in IAD (up to 50% in the case of electrocardiograms). Those results call to caution when interpreting IAD estimates. Audits based on medical records, on the local level, and an interpretation taking into account information external to IAD, on the central level, are needed to assess a more comprehensive compliance with standards.

## Introduction

A recent comprehensive report on healthcare quality in Italy published by the Organization of Economic Co-operation and Development (OECD) recommended that regular monitoring of quality of primary care by means of compliance with standards of care for chronic diseases is performed Italian National Healthcare System (NHS). Indeed, strengthening the national quality governance model on this sector of health care is a strategic objective in an ageing population, with an expected growing burden of chronic conditions. In the report, smarter payment systems for general practitioners that reward quality are advocated for, with specific reference to compliance with standards of care for chronic conditions [1].

However, measuring compliance with standards of care for chronic diseases is a challenging task for the Italian NHS [2]. Italian administrative databases (IAD) are available to the NHS uniformly from the whole country, and are the natural candidate data source. Unfortunately their use is hampered by two problems: accuracy in identifying patients who have a chronic disease, and accuracy in detecting compliance.

As for the first problem, the data items collected in IAD do not allow direct identification of patients with chronic conditions. Indeed, diagnoses performed in an outpatient setting are not collected in IAD, and this is generally the case when a chronic disease is diagnosed [3]. In Italy every adult patient is entitled to choose a general practitioner (GP), and specialist care can be requested to the NHS by patients only upon referral by their GPs. GP’s soon become aware if a chronic disease is diagnosed in their patients. Primary care medical records (MR) rather than IAD may be the right source of information to correctly identify patients having a chronic disease.

On the other hand, for the second problem, compliance with standards of care may go undetected *both* by IAD and by MR. Over-the-counter purchase of prescribed drugs is not recorded in IAD, and drug prescriptions issued by specialists are not recorded by GPs. Diagnostic tests ordered by GPs or specialists are only recorded by IAD if they are performed in facilities belonging to, or contracted by, the NHS. This may fail to happen when access to such facilities is perceived as slow or cumbersome by patients and tests are performed outside the NHS system. Diagnostic exams are recorded in the MR either if GPs are the prescribers or if patients themselves provide the result to their GPs, since there is no automatic transmission of test results in place in Italy. Ordering of diagnostic tests may more often be done by a specialist for more severe patients, or when the local organization of the healthcare system fails to encourage patients to access primary rather than secondary care. Hence sensitivity of MR in detecting diagnostic tests depends both on patient-level and on geographic-level characteristics [2].

For the reasons provided above, it was unclear whether compliance with standards of care in a population of patients with chronic diseases could be reliably estimated using IAD and, as a consequence, could be used to inform discussions on quality improvement and accountability on a local and central level. It was however evident that comparison with primary care MR had a chance to provide more knowledge on these questions [2,3,4,5].

In previous studies, case-finding strategies in IAD have been developed and validated [3], and compliance with standards of care measured on those patients has been compared with indicators obtained from a database of MR [2]. The results were encouraging, because estimates were very similar across the two data sources. However, the comparison was an ecological study, and many questions remained. It was not known whether the sample of patients detected by IAD was representative of the true set of patients with the disease, or rather patients not detected by IAD had different values of compliance. This could have combined with incompleteness of both IAD and MR in detecting actual compliance, to provide a falsely reassuring similarity between estimates.

The MATRICE Project, started by Italian National Agency for Regional Health Systems in 2011, aimed to assess in a more comprehensive way the validity of IAD as a data source to monitor quality of health care for chronic diseases. MATRICE obtained from the National Authority for Personal Data Protection permission to link IAD and MR of a large sample of patients. In this study we could therefore compare compliance with standards of care for type 2 diabetes mellitus (T2DM), hypertension and ischaemic heart disease (IHD) using both data sources, at the individual level.

## Methods

### Study design

For each person in the study population we searched both IAD and MR to determine whether they had each disease and whether they received care compliant with our standards. Based on previous study, we considered MR to be a gold standard for the presence of each disease [4,5]. Based on the arguments in the Introduction, we considered that neither IAD nor MR had complete information on compliance, so we assessed concordance among the two variables, in the whole study population. To assess concordance among measures of indicators, and representativeness of the patients that IAD correctly identified as having the disease, we compared the indicators (proportion of patients who were compliant) in several subpopulations (patients with the disease according to IAD, patients with the disease according to MR, patients that IAD correctly identified as having the disease, patients not detected by IAD, patients that IAD only classified as having the disease) using to detect compliance in turn IAD, MR and either of the two sources.

See the subsection “Study variables” below for more details on how the variables were defined, and the subsection “Data analysis” for more details on the statistical analysis.

### Setting

From the point of view of organization of health care, Italy is divided into 21 regions, and each region is divided in geographic subareas (on average 10 per region). Health care for the population in each area is managed by organizations called Local Health Units (LHU). LHUs collect administrative data on the health care they provide to their inhabitants which together form the basis of the IAD.

A sample of 25 GPs belonging to five regions was initially recruited in this study. Three regions were in the North (Lombardy, Veneto, Emilia-Romagna), one in the Center (Tuscany) and one in the South (Puglia). The GPs were equally distributed among the five regions, and all the GP’s of the same region belonged to the same LHU.

### Standards of care and indicators of compliance

A panel of experts in organization of primary care services, epidemiologists and clinicians selected clinical guidelines for T2DM, hypertension and IHD which were expected to be easy to monitor on IAD. The result is depicted in Table 1: six recommendations for annual diagnostic follow-up and treatment with four drug classes were chosen, each applying to one or more of the three conditions, totalling 18 recommendations. Each recommendation is labelled with the name of the scientific society who published it, and with its grade and level [6].

**Table 1:** Standards of care, with levels and grades of recommendation. SID: Italian Diabetes Society. ESC/EASD: European Society of Cardiology and European Association for the Study of Diabetes. ESH/ESC: European Society of Hypertension and European Society of Cardiology. ACC/AHA: American Cardiology Association and American Heart Association. A symbol * means that the recommendation only applies when the condition is at a high level of severity. Diagnostic tests are recommended once per year, except HbA1c for T2DM which is recommended twice a year.

Guideline type	Recommendation in the guideline	T2DM	Hypertension	IHD
Therapeutic	Statins	level I, grade A **(SID)**		Level IIa, grade B **(ACC/AHA)**
	Beta-blockers			Level I, grade A **(ACC/AHA)** *
	ACE inhibitors			Level I, grade A **(ACC/AHA)** *
	Antithrombotics			Level I, grade A **(ACC/AHA)**
Diagnostic	Microalbuminuria test	level VI, grade B **(SID)**	level I, grade B **(ESH/ESC)**	
	Glycated hemoglobin (HbA1c) tests	level VI, grade B **(SID)**	**level I, grade B (ESH/ESC)**	Level I, grade A **(ESC/EASD)**
	Lipid profile	level III, grade B **(SID)**	**(ESH/ESC)**	Level III, grade B **(ACC/AHA)**
	Clearence/creatinine test	level VI, grade B **(SID)**	level I, grade B **(ESH/ESC)**	
	Electrocardiogram(ECG)		level I, grade B **(ESH/ESC)**	*
	Eye exam	level III, grade B **(SID)**	level IIa, grade C **(ESH/ESC)**	

* the recommendation only applies when the condition is at a high level of severity

Based on the recommendations, 18 indicators were chosen, each measuring the proportion of patients with the specific disease who were compliant with the corresponding recommendation during the observation year. The indicators are listed in Box 1.

**Box 1:** Indicators of compliance with standards of care during a year of follow-up. **ATC**: Anatomical Therapeutic Chemical classification system for drugs. ICSO: Italian coding system for outpatient services.

Therapies
Proportion of patients with T2DM with at least two records of statins during the year (ATC: C10*) Proportion of patients with IHD treated with at least two records of statins during the year (ATC: C10*) Proportion of patients with IHD treated with at least two records of beta-blockers during the year (ATC: C07*) Proportion of patients with IHD treated with at least two records of ACE inhibitors during the year (ATC: C09*) Proportion of patients with IHD treated with at least two records of antithrombotics during the year (ATC: B01A*)
Diagnostic follow-up
Proportion of patients with T2DM with at least a microalbuminuria test (ICSO: 90.33.4) during the year Proportion of patients with hypertension with at least a microalbuminuria test (ICSO: 90.33.4) during the year Proportion of patients with T2DM with at least two tests of glycated emoglobin (ICSO: 90.28.1) during the year Proportion of patients with hypertension with at least a glycated emoglobin test (ICSO: 90.28.1) during the year Proportion of patients with IHD with at least a glycated emoglobin test (ICSO: 90.28.1) during the year Proportion of patients with T2DM with at least a test of lipid profile (ICSO: 90.14.3 and 90.14.1 and 90.43.2) during the year Proportion of patients with hypertension with at least a test of lipid profile (ICSO: 90.14.3 and 90.14.1 and 90.43.2) during the year Proportion of patients with IHD with at least a test of lipid profile (ICSO: 90.14.3 and 90.14.1 and 90.43.2) during the year Proportion of patients with T2DM with at least a test of clearence/creatinine (ICSO: 90.16.3 or 90.16.4) during the year Proportion of patients with hypertension with at least a test of clearence/creatinine (ICSO: 90.16.3 or 90.16.4) during the year Proportion of patients with hypertension with at least an electrocardiogram (ICSO: 89.52) during the year Proportion of patients with IHD with at least an electrocardiogram (ICSO: 89.52)during the year Proportion of patients with T2DM with at least an eye exam (ICSO: 95.09.1 or 95.02, or a record of an ophthalmic visit ICSO: 89.7 or 89.01 or 89.07 or 89.03, with specialty code 034), during the year Proportion of patients with hypertension with at least an eye exam (ICSO: 95.09.1 or 95.02, or a record of an ophthalmic visit ICSO: 89.7 or 89.01 or 89.07 or 89.03, with specialty code 034), during the year

### Data sources

#### Italian administrative databases (IAD)

The main components of IAD are the following tables

- Inhabitant registry (PERSON) is the list of subjects who live in a defined geographical area, recorded with gender, date of birth, date of entry, date of exit, identifier of the chosen GP;
- Hospital discharge records (HOSP) is the table of hospital discharge records reimbursed by the healthcare system, recorded with up to six diagnosis codes and up to six procedure codes in ICD9CM
- Exemption registry (EXE) is the table of disease-specific exemptions from co-payment to the healthcare system, recorded with a disease code which is a truncated ICD9CM code
- Drug dispensing registry (DRUGS) is the table of drugs dispensed by community or hospital pharmacies free of charge or upon co-payment. Drugs are coded with a specific Italian coding system, which is mapped to the Anatomical Therapeutic Chemical classification system (ATC);
- Outpatient services registry (OUTPAT) is a list of outpatient activities dispensed by the healthcare system free of charge or upon co-payment, among which specialist encounters (with no diagnostic code), laboratory or instrumental or bio-imaging diagnostic tests (without results), recorded with a specific Italian coding system

Within a LHU, all the tables above can be linked with each other at the individual level, using the national fiscal identifier as a common key. Collection of IAD tables is mandatory by national law.

#### Primary care medical records

The National College for General Practitioners (SIMG) is the national scientific society of General Practitioners (GPs) in Italy. SIMG has trained the GPs to improve the quality of recording in their medical records. In this type of medical records every visit is recorded and all diagnoses, prescriptions and measurements are recorded as part of a general practitioner’s daily practice. More than 900 members of SIMG use the same clinical software and share their de-identified medical records in Health Search, a database which is regularly used for epidemiological, public health and health services research [2, 4, 7, 8]. The GP’s belonging to the sample of this study were all participating in Health Search.

### Data collection

A script was developed by SIMG to automatically query the medical records of the 25 GPs. The script first identified all subjects in charge to the GP at 1^st^ January 2012. Then it computed variables estimating compliance with the standards of care during 2012 for each subject. Finally it applied validated algorithms to detect whether subjects had one or more of the diseases under study [4].

All the IAD data available to the healthcare system on the same population was extracted from the LHUs, using TheMatrix. TheMatrix is an open source software tool that simplifies the execution of personalized scripts on csv data [9].

Personal identifiers were pseudonymized at extraction, using the same encryption key, and all the data was automatically transmitted to the National Research Council (CNR), which had been granted permission to store and process this data. Investigators from Agenzia regionale di sanità della Toscana (ARS) developed a script to compute the study variables from IAD data, and CNR ran it on the IAD data. Finally, CNR linked the analytical dataset and medical records at individual level and transmitted the resulting dataset to ARS for statistical analysis.

One of the GPs from Lombardy was on leave in 2012 and was therefore discarded from the study after data collection.

### Study variables

Case-finding algorithms to identify patients with T2DM, hypertension and IHD from MR were selected based on a previous validation study. This study proved that the case-finding algorithms of the three diseases all had almost perfect positive predictive value [4]. Since population prevalence estimated with those algorithms is very high, sensitivity must be very high as well [3]. For this reason, in this study we used the lists of patients detected by MR as a perfect identification of the true lists of patients who should comply with the recommendations in Table 1. Case-finding algorithms to identify patients from IAD used a combination of diagnosis from hospital discharge records, disease-specific exemptions from copayment, and utilization of treatments: to detect patients who had the disease at 1st January 2012, data from HOSP, EXE and DRUGS were collected for the previous, respectively, 4, 3 and 2 years. These algorithms are described in detail in S1 Table. Sensitivity and positive predictive values of those algorithms were estimated in a separate study [5].

Compliance was defined similarly across the two data sources. The patient was considered to be compliant with a treatment if at least two records of the treatment with different dates were found in 2012, and compliant with a recommended diagnostic test if at least one prescription for that test was found in 2012, except in the case of glycated hemoglobin where two records were requested.

As discussed in the Introduction, both IAD and MR have imperfect sensitivity in detecting compliance. For each standard of care we analysed three different variables: compliance as measured by IAD, compliance as measured by MR, compliance as measured by either source (EITHER).

### Measures of compliance

For each person in the study population we had variables estimating whether the person had each disease and variables estimating whether the person was compliant with each standard, all computed both from IAD and from MR. We were therefore able to compare three ways of estimating the proportion of patients with each disease who were compliant with each standard: based on IAD only, based on MR only, and based on either IAD or MR. When based on IAD only: both patients and compliance were estimated from IAD. This is mainly the perspective from the national and regional NHS policy maker, who have only IAD data available. When based on MR only, the patients and compliance both are estimated from MR. This is usually the perspective of the GP when evaluating his/her own practice. A third perspective takes the whole set of services used by the population into account. This perspective, the true value of compliance with the standard of care in the population with the disease, is often lacking. With our data we could estimate this measure by selecting the patients with the disease from MR and the compliance from either MR or IAD: MR is the best possible population of patients, because it is a gold standard, and “either MR or IAD” is the best possible variable for compliance, because the two data sources compensate each other’s incompleteness. We refer to this as the “best possible estimate”.

### Quality governance scenarios

We considered two scenarios of quality governance where the results of our comparison can be useful, as shown in Table 2.

**Table 2:** Scenarios of quality governance where the results from this study can be used. IAD: administrative databases, MR: primary care medical records, EITHER: either among IAD or MR.

**Quality governance scenarios**		Local		Central	
**Activity**		Quality improvement		Quality monitoring	
**Comparison**	**Actors**	Local decision-makers	GPs	Local (regional) decision-makers	Regional (national) decision-makers
	**Clusters**	Patients assisted by the same GP		Patients assisted by the same LHU (region)	
	**Point of view**	Healthcare system	GP	Healthcare system	Best estimate
	**Data source used to detect patients**	IAD	MR	IAD	MR
	**Data source used to assess compliance**	IAD	MR	IAD	EITHER

In the *local* scenario, local or regional decision makers discuss quality of care with GPs with a focus on quality improvement. In this scenario, we hypothesised that the clusters of interest were the clusters of patients assisted by a same GP, and the measures to be compared were the point of view of the healthcare system (the proportion of those who compliant according to IAD among those who have the disease according to IAD) with the point of view of GP’s (the proportion of those who are compliant according to MR among those who have the disease according to MR).

In the *central* scenario, regional or national decision makers discuss quality of care, respectively, with local or regional decision makers with a focus on quality monitoring. In this scenario we hypothesised that the clusters of interest were the clusters of patients assisted by the same LHU, and the measures to be compared were the point of view of the healthcare system (the proportion of those who are compliant according to IAD among those who have the disease according to IAD) with the best estimate of the true value of the indicators (the proportion of those who are compliant according to either IAD or MR, among those who have the disease according to MR).

### Data analysis

For each disease we identified the list of patients who had the disease according to IAD, and using MR as a gold standard we marked those whose condition was unconfirmed by MR: this way we created both the list of patients that IAD correctly identified as having the disease, and the list of patients that IAD only classified as having the disease. Moreover, we identified from MR patients that were not detected by IAD. We computed the excess of true cases with respect to those in IAD who were confirmed by MR, using the formula

(Npatientswhoreallyhadthedisease/NpatientsthatIADcorrectlyidentifiedashavingthedisease)-1

Since we didn’t have a gold standard for compliance, we computed Cohen’s kappa between the compliance detected by MR and the compliance detected by IAD. Concordance was categorized as “Poor” (<0.20), “Fair” (0.21–0.40), “Moderate” (0.41–0.60), “Good” (0.61–0.80), “Very good” (0.81–1.00) [10]. Moreover, we computed the percentage of the population compliant in one source that overlapped with those compliant in the other source. We computed the increase in the compliant population when we added MR to IAD, using the formula

((NcompliantaccordingtoeitherIADorMR)/NcompliantaccordingtoIAD)-1

For each recommendation and each cluster (GP or LHU), the different measures to compute the proportion of patients who were compliant to the recommendation (Listed in the last two rows of Table 2) were standardised per age and gender, using as a standard the weights listed in S2 Table, which were computed from the age and gender distribution obtained from MR.

We estimated average difference between the indicators computed by pairs of sources on each cluster and tested significance. Estimates were obtained by fitting logistic models on a dataset with a record per patient and source, with the source of information (IAD vs MR, or IAD versus EITHER) as a dependent variable. Variance was estimated by clustering the observations on the same subject. Models were adjusted per LHU, age band and gender, with interaction between source and cluster variable (GP or LHU). In a sensitivity analysis, to test the robustness of the “best estimated” with respect to the assumption that patients detected by IAD but unconfirmed by MR didn’t have the disease, we repeated the analysis and included the patients unconfirmed by MR in the “best estimate”.

For each disease, to assess whether patients detected by IAD (both confirmed by MR and unconfirmed) were representative of the population whose disease was assessed in MR, we estimated the difference between compliance computed on patients correctly detected by IAD and, respectively, patients not detected by IAD and patients only detected by IAD. In this analysis compliance was estimated with EITHER, and was adjusted per LHU, age and gender.

### Ethics

Permission to perform record linkage between pseudonymized administrative data and medical records was granted by the Italian National Authority for the Privacy regulation. Specifically, permission was granted to CNR to store and process the data, and to ARS to obtain the linked individual-level analytical dataset, for statistical analysis.

## Results

### Study population

Data on 32,688 subjects was collected. The average number of patients per GP was 1,362 (IQ range: 1,209–1,500).

### Comparison of variables detecting diseases

**Fig 1:**
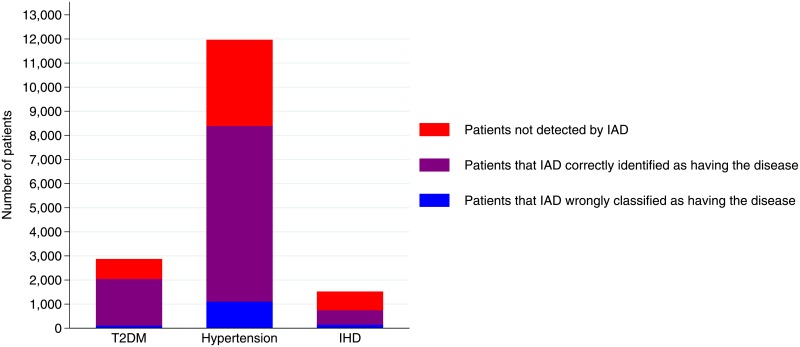
Patients detected by IAD or by MR, for each disease. “Patients that IAD only classified as having the disease” were those detected by IAD, but not by MR. "Patients that IAD correctly identified as having the disease" were identified by both IAD and MR. “Patients not detected by IAD" were identified by MR, but not by IAD.

12,673 subjects had at least one of the three diseases according to at least one of the two data sources. According to IAD, 2,047 subjects had T2DM: only 107 (5%) were patients that IAD only identified as having the disease, but additional 823 subjects (+40%) were patients having the disease, according to MR, but not detected by IAD. 8,392 subjects had hypertension according to IAD: 1,103 (13%) were patients that IAD only identified as having the disease, and additional 3,573 subjects (+42%) were patients having the disease, according to MR, but not detected by IAD. 745 subjects had IHD according to IAD: 145 (19%) were patients that IAD only identified as having the disease, and additional 776 subjects (+ 104%) were patients having the disease, according to MR, but not detected by IAD. In Fig 1 the number of patients detected by the two sources for each disease is depicted.

### Comparison of variables measuring compliance

On the general population Cohen’s kappa showed very good concordance (from 0.92 to 0.89) in the four indicators of compliance with therapies. Among diagnostic tests, concordance was very good (0.84) for microalbuminuria, good (from 0.76 to 0.66) for glycated hemoglobin, lipid profile and creatinine, moderate (0.44) for ECG and fair (0.27) for eye exams (0.27) (Table 3). Information provided by MR was almost complete (from 97% to 94%) for compliance with therapies, and was more complete than IAD in all the other indicators except eye exam (20%) (Table 3). Adding EITHER to IAD increased the measure of compliance by less than 15% in the case of therapies and of eye exam, from 24% to 32% in microalbuminuria, glycated hemoglobin, creatinine and lipid profile, and more than 50% for ECG.

**Table 3:** Comparison of compliance measured by IAD, by MR or by either of the two data sources, on the whole population. Difference in the value of indicators between the patients that IAD correctly identified as having the disease and patients not detected by IAD (ND), and between the patients that IAD correctly identified as having the disease and patients that IAD only classified as having the disease (FD). Difference was computed using EITHER for compliance, and adjusting per age, gender and LHU. Standards are listed in decreasing order of Cohen’s kappa.

					Difference in indicators between the patients that IAD correctly identified as having the disease and patients not detected by IAD (ND), and between the patients that IAD correctly identified as having the disease and patients that IAD only classified as having the disease (FD)					
Recommendation	Cohen’s K	Percentage of the population compliant in one source that overlaps with those compliant in the other source.		Percentage increase in compliance when adding EITHER to IAD	T2DM		Hypertension		IHD	
		Of those compliant according to IAD	Of those compliant according to MR		ND	FD	ND	FD	ND	FD
Statins	0.92	97.0%	89.4%	+11.5%	-19.3	-18.5			-24.6	-18.2
Betablockers	0.91	95.7%	88.8%	+12.1%					-21.9	-16.2
ACE inhibitors	0.90	96.6%	88.0%	+13.2%					-11.7	-6.9
Antithrombotics	0.89	94.5%	86.6%	+14.7%					-15.0	-12.8
Microalbuminuria	0.84	93.3%	78.1%	+26.2%	-38.6	-29.0	-13.3	-16.8		
Glycated hemoglobin	0.76	77.3%	76.6%	+23.7%	-40.1	-32.8	-4.8	-10.3	-6.7	-4.2
Lipid profile	0.73	91.0%	73.9%	+32.1%	-26.6	-13.5	-13.9	-16.3	-9.1	-2.2
Creatinine	0.66	84.2%	74.7%	+28.5%	-15.8	-13.9	-7.7	-13.9		
ECG	0.46	52.1%	50.8%	+50.5%			-5.9	-12.3	-6.5	-6.3
Eye exam	0.27	20.1%	62.5%	+12.1%	-20.1	-14.8				

### Comparison of indicators of compliance

**Fig 2:**
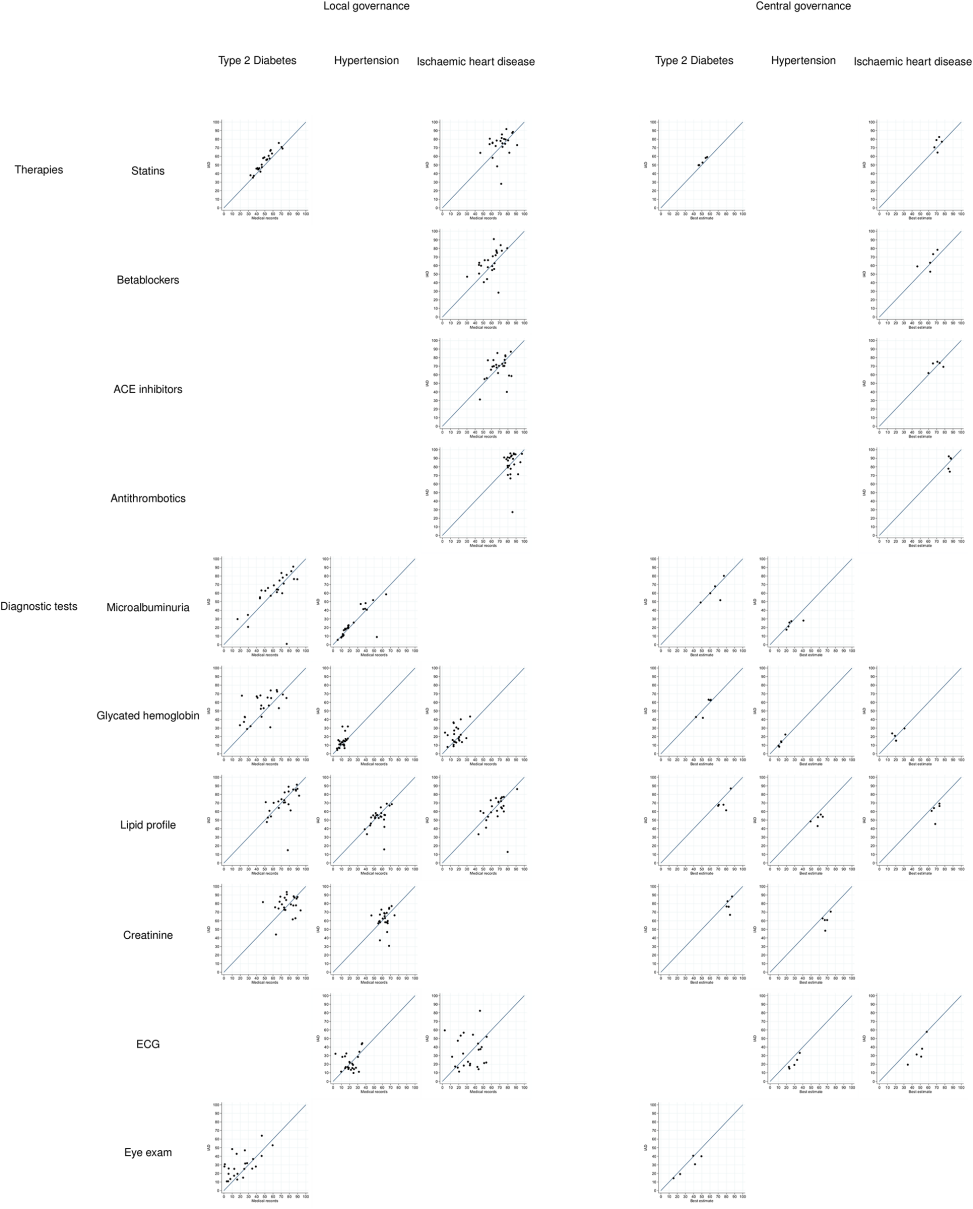
Scatter plots comparing age-and- gender standardised measures of compliance with standards of care, in the two governance scenarios. In the Local governance scenario the 24 clusters of patients of the same GP are measured by IAD on the Y-axis and MR on the X-axis. In the Central governance scenario the 5 clusters of patients in the same LHU are measured by IAD on the Y-axis and best estimate (proportion of patients detected by MR with are compliant according to EITHER) on the X-axis.

Scatter plots of the age-and-gender standardized indicators on the clusters of patients are represented in Fig 2. The corresponding values are listed in S1 File.

IAD and MR had on average very similar estimates for therapeutic indicators, although for statins in both T2DM and IHD, and for betablockers in IHD, IAD had a significantly higher estimate (respectively +4.1, +4.5 and +5.4). The results were confirmed when comparing IAD with the “best estimate”, and differences were reduced. In the case of diagnostic indicators, the picture was more complex, with IAD showing higher values than MR and lower values than the “best estimate”, often significantly. Average difference between IAD and MR was significant and higher than 5 percentage points for glycated hemoglobin and eye exam in T2DM. Average difference between IAD and “best estimate” was significant in all indicators except glycated hemoglobin, and in all but microalbuminuria and eye exam (Table 4).

**Table 4:** Average difference between the indicators computed in pairs of sources. On the left (local governance scenario): comparison between MR and IAD. On the right (central governance scenario): comparison between IAD and “best estimate”. For each indicator the p-value of the significance of the difference is shown.

		Local governance scenario						Central governance scenario					
		T2DM		Hypertension		IHD		T2DM		Hypertension		IHD	
	Indicator	Δ	p	Δ	p	Δ	p	Δ	P	Δ	p	Δ	P
Therapeutic	Statins	4.1	<0.001			4.5	<0.05	3.3	<0.001			3.6	<0.05
	Betablockers					5.4	<0.001					3.3	<0.05
	ACE inhibitors					2.2	0.152					0.0	0.992
	Antithrombotics					1.3	0.345					-0.5	0.691
Diagnostic	Microalbuminuria	-0.1	0.931	-0.2	0.543			-3.4	<0.001	-2.3	<0.001		
	Glycated hemoglobin	8.9	<0.001	2.2	<0.001	3.0	<0.05	-0.1	0.929	-0.0	0.950	-1.1	0.366
	Lipid profile	-3.0	<0.001	-3.9	<0.001	-2.9	0.074	-6.3	<0.001	-7.2	<0.001	-6.9	<0.001
	Creatinine	0.8	0.402	-1.2	<0.05			-5.7	<0.001	-7.4	<0.001		
	ECG			0.1	0.891	1.2	0.539			-7.7	<0.001	-14.1	<0.001
	Eye exam	7.5	<0.001					-4.7	<0.001				

The sensitivity analysis confirmed the results obtained in the comparison between IAD and the “best estimate”: the estimates changed by around one percentage point (S3 Table).

### Representativeness of subpopulations

Indicators in patients not detected by IAD were much lower (from -15.8 to -40.1 percentage points difference) with respect to indicators in patients correctly detected by IAD in the case of T2DM, and substantially lower (from -6.7 to -24.6 percentage points difference) in the case of IHD (Table 3). They were lower in the case of hypertension, too, but less so (from -4.8 to -13.9 percentage points difference). Differences were higher for indicators of therapies. Differences between patients detected by IAD and confirmed by MR and patients only detected by IAD were similar but slightly smaller (Table 3).

## Discussion

Measures of compliance in the whole population were concordant between MR and IAD in the case of therapies, less so in the case of diagnostic testing, especially when more complex tests were considered (ECG and eye exam). Indicators of compliance with therapies showed low average difference between data sources, although still significant in some cases. Indicators of compliance with diagnostic monitoring were imbalanced: IAD estimated higher compliance with respect to MR, and lower compliance with respect to the best possible estimate. This was the result of a combination of different errors. Patients detected by IAD were not representative of the true population of patients, especially in the case of T2DM and of therapeutic indicators. Small average differences between the estimates of IAD and the best estimates were partially coincidental and therefore run the risk of not being reproducible in all the regions and across time.

### Interpretation of the findings: Estimating compliance with recommended therapies and diagnostic tests

This individual-level study showed that the confounding effects anticipated in the limitations of the ecological study were indeed playing an important role in the estimate of indicators performed on IAD.

The effects of different misclassifications (of patients and of compliance) were balanced in the case of therapies, because concordance between MR and IAD was high, and MR was almost complete; therefore, the absence from the denominator of the indicator of those patients who were not detected by IAD, who had lower compliance, compensated the small overall underestimation of the compliance. A small contribution to the balance was also provided by the comparatively small share of patients that IAD only classified as having the disease, who had similar compliance profile as patients not detected by IAD.

As expected, measures of compliance obtained from IAD and MR were less concordant in the case of diagnostic tests, and compliance measured by IAD was lower. The combination of errors produced both balanced and imbalanced results. In the case of glycated hemoglobin test, in the patients not detected by IAD the indicator was less that 40 percentage points lower than in patients correctly identified by IAD as having the disease and the overall agreement between the administrative and “best estimate” was due to underestimation of compliance on the patients detected by IAD. However there was an important imbalance between IAD and MR estimate in T2DM patients. In recent, similar validation studies of estimates of measures of performance on diabetic patients from administrative databases from the United States, similar mixed effects were observed [11, 12].

In the scatterplots in Fig 2, IAD measured a very low compliance with Microalbuminuria and Lipid profile indicators on a GP, in the patients of all the three diseases. Those patients were all assisted by the same GP (see S1 File), and it is therefore possible that some local issue, such as the absence of a nearby laboratory contracted with the NHS, or a systematic mistake made by a local laboratory in coding those specific exams, was at the root of this finding. As a consequence, in the same indicators IAD measured lower compliance than MR in the LHU of this GP. In the same figure, IAD measured low compliance with Statins, Betablockers and Antithrombotics treatment in patients with IHD of a GP. The GP was the same in the three cases, but different with respect to the outlier GP in the indicators of compliance with diagnostic recommendations (see S1 File). The outlier GP of therapeutic indicators in IHD was also the one with lowest number of IHD patients detected by IAD (15 patients). In this case, chance may have played a role in concentrating among the half of the patients of this GP detected by IAD a higher socio-economic status, that may have resulted in a higher proportion of such patients purchasing drugs out-of-pocket. As an alternative explanation, the local pharmacy may have transmitted incomplete or corrupted records to the LHU.

### Consequences on the use of Italian administrative data in a systematic quality monitoring and improvement strategy

In a quality monitoring strategy IAD is a reliable tool for signalling purposes: when IAD detects either an excellent or a poor performance in a cluster of patients, according to our data it is very likely that the observation is correct, particularly in the case of compliance with therapeutic standards, and with yearly laboratory diagnostic tests.

However, we found that a combination of mutually balancing misclassifications is at the root of the similarity between IAD results and our best estimate of the true compliance in the patients with a diagnosed chronic disease, especially in the case of diagnostic recommendations. Specific caution should be taken in interpreting coverage of the twice-yearly glycated hemoglobin test in diabetic patients. Likewise, the measures of compliance with annual eye exam in diabetics, and annual ECG in hypertensive and IHD patients look fragile.

This has slightly different implications for a “local” quality improvement rather than a “central” quality monitoring scenario.

In a local scenario the main actors are, on the one hand, the local (or regional) decision-makers for the organization of healthcare for chronic diseases and, on the other, the GPs. The main objective is promoting appropriateness in healthcare for chronic diseases, that is, supporting the role of primary care as the main driver, in close collaboration with specialist care [1]. Thanks to IAD, decision makers have the possibility of producing estimates of compliance across a range of GPs. While this sort of comparison is in itself very informative, it is clear from our validation that it is not precise enough to provide a reliable ranking of the performance of GPs, nor to support quality-based payment systems, such as a pay-for-performance scheme. Rather it should be taken as the starting point for quality improvement initiatives, such as a more detailed audit of quality based on medical records. Clusters of patients with low compliance, as signalled by IAD, must be analysed in conjunction with context information, such as local issues in data quality, accessibility to local NHS facilities for diagnostic tests, and possible drive of local specialist healthcare providers towards replacing, rather than supplementing, primary care, sometimes implying out-of-pocket purchase of care. All those elements can provide input to action for local decision-makers. Clusters with high compliance, in turn, must be critically analysed: if patients with mild forms of chronic diseases are not appropriately followed-up, they will remain undetected by IAD, which will therefore measure higher compliance only on the more severe patients, thus providing a falsely reassuring picture. This is likely to be associated with clusters where IAD detects low prevalence. Aside from those extremes, quality governance at the local levels should focus on an integrated interpretation of IAD and MR data, which are both available to the actors.

In a central scenario the main actors are all decision-makers for the organization of healthcare system, at different levels: local vs regional, or regional vs national. The main objective is monitoring quality of healthcare and making comparisons between the different geographical entities to assure equity in quality of care amongst the whole Italian population. Integrated analysis of IAD and MR is not possible in this scenario, therefore context for interpretation of signals from IAD must be carefully built in collaboration with local decision-makers, who can provide crucial context information, in particular findings from local analysis of MR. Several resources are available to inform this assessment: SIMG produces a yearly report comparing compliance with standards of care across Italian regions estimated from MR of a sample of GPs belonging to the Health Search network [13], and survey data are produced every five years by the National Institute of Statistics, estimating access to NHS specialist facilities [14].

### Developments

The proportion of patients not detected by IAD was substantial, and this finding has consequences that go beyond the objective of this study. A more extensive treatment of this issue can be found in a separate study.[5]

Routine data-linkage between administrative data and key elements from primary care medical records, such as diagnosis of a chronic disease and compliance with standards of care, would critically improve the quality governance of primary care. Local initiatives have been initiated to this respect, such as the SOLE network in Emilia-Romagna [1].

Analytical calibration methods that include the results from this validation study, as well as aggregated measures produced by SIMG and the National Institute of Statistics, could be developed to improve estimates produced by IAD.

### Implications for the use of the indicators in studies of impact

Indicators of compliance with standards of care can be used to evaluate the impact of innovative strategies [15, 16, 17]. Our results support overall this use of the indicators, provided a difference-in-differences design is adopted, and the impact is measured across a short time span, so that it can be assumed that misclassification does not change differentially across exposed and non exposed to the intervention. If this is not possible, elements that may imbalance misclassification across exposed and non-exposed, or across time, need to be discussed in the limitations of the study.

### Permission to perform record linkage was an extraordinary result

This study was made possible by an explicit permission of the Italian National Authority for the Privacy regulation, which allowed individual-level record linkage between IAD and MR on a large sample of patients. It is encouraging that such permission was granted, and routes for expedited permission should be created, especially for validation studies of administrative data. Indeed, this would allow rapid generation of evidence crucial for public health and health system governance in a transparent and legal manner.

### Limitations

The variable that we used as a “best estimate” of compliance may have overestimated the true compliance, as GP drug prescriptions may have not been filled in, and GP test orders may have not been performed in reality. The first effect is however likely to be small, as a second prescription is required for the patient to be compliant, according to the algorithm we adopted in our computations. Moreover, the concordance we observed between MR and IAD data was very high when measuring compliance with therapies (Table 3).

The indicators of compliance with a recommended therapy adopted by the MATRICE panel of experts (Box 1) use a simple rule (at least two records in a year). This rule is easy to implement across different data sources, such as IAD and MR, and has been used since 2004 to compute similar indicators in the Quality and Outcome Framework of the National Health System of the United Kingdom [18,19], which have been proven to be effective for the purposes of quality monitoring and improving [18]. However, an indicator computed with this rule does not measure true adherence to therapy.

In our analysis we assumed that patients classified as having a disease by IAD, but unconfirmed by MR, were in fact without the disease. To assess robustness of results to this assumption, we first showed that the number of persons detected by IAD and unconfirmed by MR is small, in comparison to the number of people detected by MR, especially in the case of T2DM. Moreover, we tested the impact of the assumption on the "best estimate" in a sensitivity analysis, and showed that the difference is around one percentage point, which leaves unchanged the interpretation of our findings.

## Conclusion

IAD overestimated the percentage of patients compliant with therapeutic standards by less than 6 percentage points, and underestimated the percentage of patients compliant with diagnostic standards by a maximum of 14 percentage points. Therefore, both discussions at local level between GP's and local health unit managers and discussions at central level between national and regional policy makers can be informed by indicators of compliance estimated by IAD, which, based on those results, have the ability of signalling critical or excellent clusters. However, this study found that estimates are partly flawed, because a high number of patients with chronic diseases are not detected by IAD, patients detected are not representative of the whole population of patients, and some categories of diagnostic tests are markedly underrrecorded in IAD (up to 50% in the case of electrocardiograms). Those results call to caution when interpreting IAD estimates. Audits based on medical records, on the local level, and an interpretation taking into account information external to IAD, on the central level, are needed to assess a more comprehensive compliance with standards.

## Supporting information

S1 Table Case-finding algorithms for type 2 diabetes mellitus, hypertension, and ischaemic heart disease. Algorithms from hospital discharge records select diagnostic fields coded in ICD9CM. Algorithms from exemption from health care copayment select diagnostic fields coded in an Italian coding system similar to a 3-digit-truncated ICD9CM. Algorithms from drug dispensings select the ATC code of the drug.
(DOC)

S2 Table Age and gender distribution. Age and gender distribution in the standard population for type 2 diabetes mellitus (T2DM), hypertension and ischaemic heart disease (IHD).
(DOC)

S3 Table Sensitivity analysis with respect to Table 4. Average difference between the indicators: comparison between IAD and the estimate obtained from the “best estimate” by including in the denominator also patients detected only by IAD. For each indicator the p-value of the significance of the difference is shown.
(DOC)

S1 File Detailed results. Number of patients and age-standardized indicators with 95% confidence interval (CI) of all the GPs, according to IAD and to MR, and of all LHUs, according to IAD and best estimate.
(XLS)

## Abbreviations

ARS: Agenzia regionale di sanità: the institution where data analysis was conducted
ATC: Anatomical Therapeutic Chemical classification system for drugs from the World Health Organization Collaborating Centre for Drug Statistics Methodology http://www.whocc.no
CNR: National Research Council: the institution which developed the data extraction and management tool TheMatrix
ECG: Electrocardiogram
EITHER: the individual-level variable that measures compliance with a recommendation using either MR or IAD
GP: general practitioner
IAD: Italian Administrative Databases
ICSO: Italian coding system for outpatient services
IHD: Ischaemic heart disease
LHU: Local Health Unit, the local organization of the NHS
MR: Medical Records: the primary care medical records of the 24 GP's whose patients entered the study
NHS: National Health Service
OECD: Organization of Economic Co-operation and Development
SIMG: National College for General Practitioners: the Italian scientific society of General Practitioners
T2DM: Type 2 Diabetes Mellitus
