# Supplementary material for: Monitoring compliance with standards of care for chronic diseases using healthcare administrative databases in Italy: Strengths and limitations
Source: PLoS One. 2017 Dec 12;12(12):e0188377. doi: 10.1371/journal.pone.0188377 (PMC5726627; doi:10.1371/journal.pone.0188377)
Supplement: S1 Table — Algorithms from hospital discharge records select diagnostic fields coded in ICD9CM. Algorithms from exemption from health care copayment select diagnostic fields coded in an Italian coding system similar to a 3-digit-truncated ICD9CM. Algorithms from drug dispensings select the ATC code of the drug. (DOC) [file pone.0188377.s001.doc]

S1 Table. Case-finding algorithms for type 2 diabetes mellitus, hypertension, and ischaemic heart disease.

Algorithms from hospital discharge records select diagnostic fields coded in ICD9CM. Algorithms from exemption from health care copayment select diagnostic fields coded in an Italian coding system similar to a 3-digit-truncated ICD9CM. Algorithms from drug dispensings select the ATC code of the drug.

| **Type 2 diabetes mellitus** |
| --- |
| At least one hospital discharge record with a diagnosis (250*0 OR 250*2) in the previous 4 years  OR  At least one exemption from health care copayment with diagnosis *250 in the previous 3 years  OR  At least 2 dispensings in a year of insulin (A10A*) in the previous 2 years  OR  At least 2 dispensings in a year of non-insulin antidiabetic drugs (A10B*) in the previous 2 years |
| **Hypertension** |
| At least one hospital discharge record with a diagnosis ( 401* OR 402* OR 403* OR 404* OR 405* ) in the previous 4 years  OR  At least one exemption from health care copayment with diagnosis (*000 OR *401 OR *402 OR *403 OR *404 OR *405 OR 031* OR A31*) in the previous 3 years  OR  At least 2 dispensings in a year of antihypertensives (C02*) in the previous 2 years  OR  At least 2 dispensings in a year of betablockers or renin-angiotensin agents (C09* OR C07*) in the previous 2 years |
| **Ischaemic heart disease** |
| At least one hospital discharge record with a diagnosis (410* OR 411* OR 413* OR 414*) in the previous 4 years  OR  At least one exemption from health care copayment with diagnosis *414 in the previous 3 years  OR  At least 2 dispensings in a year of organic nitrates (C01DA*) in the previous 2 years |
