# Supplementary material for: Monitoring compliance with standards of care for chronic diseases using healthcare administrative databases in Italy: Strengths and limitations
Source: PLoS One. 2017 Dec 12;12(12):e0188377. doi: 10.1371/journal.pone.0188377 (PMC5726627; doi:10.1371/journal.pone.0188377)
Supplement: S2 Table — Age and gender distribution in the standard population for type 2 diabetes mellitus (T2DM), hypertension and ischaemic heart disease (IHD). (DOC) [file pone.0188377.s002.doc]

| Gender | Age Group | T2DM | Hypertension | IHD |
| --- | --- | --- | --- | --- |
| M | 16-44 YEARS | 1.8 | 3.3 | 0.4 |
| F |  | 2.2 | 2.1 | 0.2 |
| M | 45-64 YEARS | 18.3 | 18.2 | 16.5 |
| F |  | 11.2 | 16.9 | 4.1 |
| M | 65-84 YEARS | 30.4 | 22.4 | 40.8 |
| F |  | 26.6 | 27.3 | 22.0 |
| M | 85+ YEARS | 3.1 | 3.1 | 7.0 |
| F |  | 6.4 | 6.9 | 9.0 |

**S 2 Table. Age and gender distribution.** Age and gender distribution in the standard population for type 2 diabetes mellitus (T2DM), hypertension and ischaemic heart disease (IHD)
