# Supplementary material for: Monitoring compliance with standards of care for chronic diseases using healthcare administrative databases in Italy: Strengths and limitations
Source: PLoS One. 2017 Dec 12;12(12):e0188377. doi: 10.1371/journal.pone.0188377 (PMC5726627; doi:10.1371/journal.pone.0188377)
Supplement: S3 Table — Average difference between the indicators: comparison between IAD and the estimate obtained from the “best estimate” by including in the denominator also patients detected only by IAD. For each indicator the p-value of the significance of the difference is shown. (DOC) [file pone.0188377.s003.doc]

**S 3 Table. Sensitivity analysis with respect to Table 4.** Average difference between the indicators: comparison between IAD and the estimate obtained from the “best estimate” by including in the denominator also patients detected only by IAD. For each indicator the p-value of the significance of the difference is shown.

|  |  | T2DM | | Hypertension | | IHD | |
| --- | --- | --- | --- | --- | --- | --- | --- |
|  |  | Δ | P | Δ | p | Δ | P |
| Therapeutic | Statins | 3.6 | <0.001 |  |  | 4.9 | <0.001 |
|  | Betablockers |  |  |  |  | 4.5 | <0.001 |
|  | ACE inhibitors |  |  |  |  | 0.8 | 0.521 |
|  | Antithrombotics |  |  |  |  | -0.1 | 0.965 |
| Diagnostic | Microalbuminuria | -2.3 | <0.05 | -1.2 | <0.001 |  |  |
|  | Glycated hemoglobin | 0.8 | 0.319 | 0.1 | 0.753 | -0.6 | 0.555 |
|  | Lipid profile | -5.6 | <0.001 | -6.3 | <0.001 | -6.2 | <0.001 |
|  | Creatinine | -5.2 | <0.001 | -7.0 | <0.001 |  |  |
|  | ECG |  |  | -7.4 | <0.001 | -13.5 | <0.001 |
|  | Eye exam | -4.2 | <0.001 |  |  |  |  |
